# Quality of care for tuberculosis patients in public health facilities of Debre Tabor town, Northwest Ethiopia

**DOI:** 10.1371/journal.pone.0234988

**Published:** 2020-06-19

**Authors:** Chalachew Genet, Tesfaye Andualem, Addisu Melese, Wondemagegn Mulu, Feleke Mekonnen, Bayeh Abera

**Affiliations:** 1 Department of Medical Laboratory Sciences, College of Medicine and Health Sciences, Bahir Dar University, Bahir Dar, Ethiopia; 2 Department of Medical Laboratory Science, College of Health Sciences, Debre Tabor University, Debre Tabor, Ethiopia; The University of Georgia, UNITED STATES

## Abstract

**Background:**

Even though there are different tuberculosis (TB) prevention and control measures implemented globally including Ethiopia, TB is still major public health problem. This is partly due to compromised quality of care delivered for tuberculosis patients in health facilities (HFs) during diagnosis, treatment and follows-up. Thus this study is intended to determine the quality of care delivered for TB patients in all public HFs of Debre Tabor town, Northwest Ethiopia.

**Methods:**

Cross sectional study was conducted from January to May 2018. Data were collected with face-to-face interview and TB registration book review using structured questionnaire and checklist respectively. Collected data was entered and processed using SPSS and P value <0.05 was considered statistically significant. The quality of care for each HF was graded as very good, good, moderate, poor and very poor if HFs achieve [90–100%], [80–90%), [70–80%), [60–70%) and <60% of performance indicators respectively using Donabedian structure, process and outcome model of health care quality.

**Results:**

All HFs have sputum collection area, enough microscopic slide, at least one functional microscope and sufficient anti TB drug supply. But HFs lack backup laboratory stains. Overall structural aspects of quality of care in all HFs were very poor achieving 42.5–52.9% structural performance indicators out of 100%. Similarly the overall process aspects of quality of care was poor in all public HFs which achieved 60–68.9% of the scores out of 100%. In the study; 68.9%, 54.5% and 80.6% of Medical Laboratory, pharmacy and other healthcare workers (HWs) provided correct response respectively on TB causative agent, risk factor, transmission, treatment, prevention, case management and case finding strategies. HWs who knew at least two TB case finding strategies in DTH was significantly higher than those HWs working in Health Centers (P = 0.004). On the other hand, except Ginbot 20 HC, HFs was graded as good by scoring 86.6–89.3% of performance indicators on the outcome aspects of quality of care. In all HFs studied, all TB patients’ unit TB registration number, sex, age, TB category, treatment initiation date and intensive phase treatment start year were properly registered. Moreover 110 (78%) and 147 (69%) contact person address in DTH and HCs was properly registered on TB unit register book respectively with no statistical difference in hospital and HCs (P = 0.063). There was proper TB patients’ address registration in hospital than HCs studied (P< 0.001).

**Conclusions:**

The outcome aspects of quality of care for TB patients in all HFs were promising. But structural & process aspects of quality of care was compromised which necessitate different corrective actions to be taken by different stakeholders to enhance quality of care for TB patients in public HFs studied. Moreover based on the study findings, continuous supply of drugs, laboratory equipment and reagents, availing current guideline/s in HFs, providing up-to-date training for HWs on TB and proper documentation are important to improve quality of care provided for TB patients.

## Introduction

Tuberculosis (TB) is curable bacterial infectious disease. Since its identification, the disease is causing significant morbidity and mortality globally. It kills around 5,000 people each day. From the total TB death, 98% of them live in developing world. In 2014, for example, there were 9.6 million new TB cases and 1.5 million TB deaths [1]. TB causes significant morbidity and mortality throughout the world. As a result, it was declared as global emergency by World Health Organization (WHO) since 1993 [2]. The impact of TB is more pronounced in developing than developed countries. In Africa, for example, 281 cases per 100, 000 population is reported in 2014 which is twice of the global average (133/100,000 population) on the same year [3].

As part of developing country, Ethiopia is highly affected by TB. Based on 2016 WHO report, the incidence of TB cases in Ethiopia in 2014 were 207 per 100, 000 population [3]. Moreover TB is the second cause of death in Ethiopia based on 2009/10 health & health related indicators of the Federal Ministry of Ethiopia [4].

Despite different prevention and control measures implemented, TB is still causing significant morbidity & mortality in Ethiopia. One of the contributing factors is compromised quality of care delivered for TB patients in different health facilities (HFs) [5–8]. But little is known in the current quality of care provided in public HFs of Debre Tabor town, Amhara regional state of Ethiopia. Thus the present study was conducted to determine the quality of care delivered for TB patients in the study area on the three aspects of quality of care (structure, process and outcome).

## Materials and methods

### Study design and setting

Health facility based cross sectional study was conducted in Debre Tabor Town of Amhara regional state, Ethiopia from January 1 to May 30, 2018. The town is located 100 km and 666 km away from regional capital Bahir Dar and country capital Addis Ababa respectively. Based on 2014 Central Statistics Agency (CSA) report, the town has a total population of 55, 596. The town has one public hospital namely Debre Tabor Hospital (DTH) and three public Health Centers (HC) namely Hidar 11 HC, Debre Tabor Health Center (DTHC) and Ginbot 20 HC. In all HFs, TB diagnosis, treatment and prevention service is given. The town also hosts different higher institutions which include Debre Tabor University, College of teacher education, Debre Tabor College of health science and others.

### Population

#### Target population

All public HFs and HWs working in public HFs of South Gondar zone.

#### Study participants

All public HFs and HWs working in public HFs of Debre Tabor town and TB patients from two year document review of the HFs TB clinic.

### Inclusion & exclusion criteria

For HWs, the following points were considered as inclusion and exclusion criteria.

#### Inclusion criteria

All HWs who worked at least for the past three months in his/her present HF as well as give written consent to participate in the study were included.

#### Exclusion criteria

Any HW who were in break leave at the time of data collection as well as those HWs who refused to give written consent.

### Sample size determination

All four public HFs in the town providing TB diagnosis, treatment and prevention service were included. All 112 HWs fulfilling the inclusion criteria and 354 TB patients from two year document review were included in the study for assessing the structure, process and outcome aspects of quality.

### Sampling technique

All public HFs found in Debre Tabor town, HWs working in the HFs and two year document review of all TB patient in all HFs from January 2016-December 2017.

### Data collection

From all four HFs, data were collected using structured questionnaire and checklist from HWs and TB registration book respectively by 4 trained BSc degree holder HWs. The structured questionnaire was developed using the national TB guideline [4] and different related literatures [7, 8, 9, 10]. Data from 112 HWs was collected to assess the structure and process aspects of quality of care. Among 112 HWs included: 72, 15, 11 and 14 were from DTH, DTHC, Ginbot 20 HC and Hidar 11 HC respectively.

All HFs used similar unit TB registration book provided by the federal ministry of health, Ethiopia. Thus the checklist, having 19 items, were developed from this unit TB registration book to collect data from all 354 TB patients who attended their treatment from January 2016-December 2017 to assess the outcome aspects of quality of TB care delivered.

### Quality control

Pre-test was done on HC found in “Woreta” town found in same administrative zone using the developed questionnaire and checklist. Moreover during data collection, every questionnaire was cheeked for its completeness just at the end of data collection.

### Data analysis

The collected data was entered and analyzed using SPSS to assess the quality of care delivered for TB patients by considering the three dimensions of quality of care based on Donabedian’s structure-process-outcome model of health care quality which was also used by different related researches done [7, 9, 11]. Different statistical analysis including frequency, percentage, Pearson’s Chi square test and Fisher’s exact test were done.

#### Operational definitions

*Structural aspects of quality*. The structural aspects of quality of care delivered was assessed and analyzed at different units of each HFs and at overall HF level. The structural aspect of health care quality delivered in laboratory unit, pharmacy unit and in other units of the HFs were assessed using 14, 10 and 8 questions/items respectively. Generally the questions included were designed to assess the availability of equipment like microscope, laboratory materials, reagents, TB guidelines, anti-TB drugs and training to health professionals. By considering the total maximum attainable score as 100%, the actual score was summed and percentage was calculated and the quality of care provided by HF/Unit was graded based on the percentages achieved (Table 1).

**Table 1:** Performance indicator used to measure the quality of care delivered for TB patients in public health facilities of Debre Tabor town in South Gondar Zone, Ethiopia.

Number of items assessed	Assigning marks for items	% achieved	HF or HF Unit Quality level
1. 32^¥^, 2. 74^§^ 3. 25^Δ^	1.1. Presence/yes response^¥^ = 1 1.2. Absence/no response^¥^ = 0 2.1. Knowing correct Response^§^ = 1 2.2. Not knowing correct response^§^ = 0 3.1. Cure/treatment complete/correctly recorded value^Δ^ = 1 3.2. Other treatment outcomes/incorrectly recorded value^Δ^ = 0	[90–100%]	Very good
		[80–90%)	Good
		[70–80%)	Moderate
		[60–70%)	Poor
		<60%	Very poor

¥→ for structure aspect of quality

§→ for process aspects of quality

Δ→ for outcome aspects of quality

*Process aspects of quality*. It was assessed and analyzed at different units of each HFs and at overall HF level. The process aspects of health care quality in laboratory unit, pharmacy unit and in other units of HFs were assessed using 23, 28 and 23 questions/items respectively. The questions were designed to assess the knowledge of HWs on TB causative agent, transmission, treatment, treatment monitoring mechanisms, prevention and control methods. By considering the total maximum attainable score as 100%, the actual score was summed and percentage was calculated and the quality of care provided by HF/Unit was graded based on the percentages achieved (Table 1).

*Outcome aspects of quality*. It was assessed and analyzed using the data collected from two year document review of unit TB register book using cheek list having 25 items including TB patient treatment outcomes. Then considering the total maximum attainable score as 100%, the actual score was summed and percentage was calculated and the quality of care provided by HF/Unit was graded based on the percentage achieved (Table 1).

*Other health workers*. A health worker other than pharmacy and ML professionals who is involved in the diagnosis, treatment, follow-up and/or prevention of TB in HFs studied.

### Ethical consideration

Before data collection, ethical clearance was obtained from Debre Tabor University institutional review board (IRB) with ethical approval number Ref. N^o^-DTU/RE/1/106-P5/24. Moreover a written consent was obtained from each study participants.

## Results

The present study assessed the structural, process and outcome aspects of quality of care delivered for TB patients.

### Structure aspects of quality

Among 112 HWs included, 54 (48.2%) were females with a mean age of 30 years and 58 (51.8%) were males with a mean age of 27.5 years. The study showed that 52.8% of HWs working in DTH had educational level of BSc degree and above unlike HWs working in Ginbot 20 health center where only 27.3% had educational level of BSc degree and above (Table 2).

**Table 2:** Educational level of health workers and their involvement on TB health education delivery in DTH and health centers of Debre Tabor town from January to May 2018.

Educational level (n = 112)	Debre Tabor Hospital: Number (%)	Debre Tabor Health Center: Number (%)	Ginbot 20 Health Center: Number (%)	Hidar 11 Health Center: Number (%)
Diploma	31 (43%)	10 (66.7%)	7 (63.6%)	5 (35.7%)
Degree and above	41 (57%)	5 (33.3%)	3 (27.3%)	5 (35.7%)
Level I-IV	0 (0%)	0 (0%)	1 (9.1%)	4 (28.6%)
**Health worker involvement on TB health education**				
No	Variables	Involved No (%)	Not involved No (%)	Total No (%)
1	Health facility	Other health workers		
	DTH	17 (81)	4 (19)	21 (100)
	HCs	13 (56.5)	10 (43.5)	23 (100)
	Significance	Fisher’s Exact Test P = 0.111		
2	Health facility	ML		
	DTH	5 (31.3)	11 (68.7)	16 (100)
	HCs	7 (63.6)	4 (36.4)	11 (100)
	Significance	Fisher’s Exact Test P = 0.130		
3	Health facility	Pharmacy		
	DTH	2 (28.6)	5 (71.4)	7 (100)
	HCs	1 (16.7)	5 (83.3)	6 (100)
	Significance	Fisher’s Exact Test P = 1		

DTH→ Debre Tabor Hospital, DCs→ Health Centers, ML→ Medical Laboratory

The present study also indicated that all HFs hadn’t had guideline for clinical and programmatic management of TB, Leprosy and TB/HIV. On the other hand all HFs had at least one TB posters which was written either in local language “Amharic” or in English. Moreover all HFs delivered health education on different topics of TB but 29 (25.9%) HWs included in the study didn’t know the existence of health education on TB in their HFs.

Among 83 (74.1%) HWs who know the existence of health education on TB in there working HF, their involvement in delivering health education on TB topics was different in different health professionals. Only 3 (20%) pharmacy workers were involved in delivering health education on TB related topics. The study also indicated that ML workers with BSc degree and above had better involvement in delivering health education than those ML workers having educational level below BSc degree (P = 0.038). But such kind of association was not observed among other HWs (P = 0.052). Working in hospital and HC did not have statistical significant association in HWs’ involvement on TB education (Table 2).

All HFs had separate sputum collection area, enough microscopic slides and at least one functional microscope to deliver laboratory diagnosis service for TB patients. Moreover three out of four HFs have enough Auramine-Phenol (A-P) staining reagents for sputum smear microscopy to diagnose TB. On the other hand, 58.1% of ML workers indicated that their HF hadn’t had enough Ziehl-Neelson (Z-N) staining as a backup for A-P staining reagent.

Moreover 81.5% and 92.6% pharmacy workers indicated that their HF had sufficient anti TB drugs for TB patients in working days and weekends respectively for three months. All HFs assessed have at least 2 HWs who had taken in-service training on DOTS. Moreover Hidar 11 HC had the highest percentage of HWs (21.4%) trained on DOTS whereas 40.3% of HWs working in DTH were trained on TB & TB related topics other than DOTS which was the highest compared with other HFs (Table 3).

**Table 3:** In-service training profile of HWs and their satisfaction on current payment and health institute management system from January to May 2018.

In-service training type	DTH: Number (%)	DTHC: Number (%)	Ginbot 20 HC: Number (%)	Hidar 11 HC: Number (%)
➢ Trained on TB guideline	10 (13.9%)	1 (6.7%)	3 (27.3%)	3 (21.4%)
➢ Trained on DOTS	14 (19.4%)	2 (13.3%)	2 (18.2%)	3 (21.4%)
➢ Trained on other topics	29 (40.3%)	5 (33.3%)	5 (45.5%)	6 (42.6%)
Health worker satisfaction				
➢ Satisfied on the payment	8 (11.1%)	3 (20%)	1 (9.1%)	4 (28.6%)
➢ Payment affect service quality	32 (44.4%)	4 (26.7%)	4 (36.4%)	4 (28.6%)
➢ Satisfied on management	31 (43.1%)	9 (60%)	6 (54.6%)	5 (35.7%)
➢ Management affect service quality	22 (30.6%)	5 (33.3%)	3 (27.3%)	6 (42.9%)

DTH→ Debre Tabor Hospital, DTHC→ Debre Tabor Health Center, HC→ Health Center

Only 7.4% had taken one or more in-service training among the three in-service trainings assessed. On the other hand 64.8% and 12.9% of other HWs and ML workers included in the study had taken at least one in-service training among the three training assessed respectively.

Among HWs interviewed, 85.7% and 54.5% were not satisfied with the current payment and their HF management system respectively. Moreover 39.3% and 32.1% of HWs believed that the current payment and management system of their HF will negatively affect the quality of care delivered for TB patients respectively (Table 3).

Generally the overall structural aspects of quality of care delivered were very poor with achieved score less than 60% out of 32 items assessed (Table 4).

**Table 4:** Structural aspect of quality of care delivered for TB patients in four public health facilities of Debre Tabor town from January to May 2018.

No	Health Facility	Unit	Structure quality attribute by unit % (No of HWs/item No)	Unit Status	Structure quality attribute by HF % (No/item No)	HF Status
1	DTH	Laboratory	55.67(20/14)	VP	42.5 (70/32)	VP
		Pharmacy	26.6 (19/10)	VP		
		OHW	39.6 (31/8)	VP		
2	DTHC	Laboratory	61.9 (3/14)	Poor	46.5 (15/32)	VP
		Pharmacy	40 (2/10)	VP		
		OHW	37.5 (10/8)	VP		
4	Ginbot 20 HC	Laboratory	59.5 (6/14)	VP	52.9 (13/32)	VP
		Pharmacy	45 (2/10)	VP		
		OHW	45 (5/8)	VP		
3	Hidar 11 HC	Laboratory	57.1 (4/14)	VP	48.1 (14/32)	VP
		Pharmacy	40% (2/10)	VP		
		OHW	39.1% (8/8)	VP		

DTH→ Debre Tabor Hospital, DTHC→ Debre Tabor Health Center, Ginbot 20 HC→ Ginbot 20 Health Center, Hidar 11 HC→ Hidar 11 Health Center, VP→ Very Poor, OHW→ Other Health Workers

### Process aspects of quality

For process aspects of quality, 23, 28 and 23 questions were used to assess knowledge of ML workers, Pharmacy workers and other HWs respectively. Correct responses were obtained from 68.9%, 54.5% and 80.6% of ML, pharmacy and other HWs respectively.

All pharmacy, ML and other HWs know TB causative agent, transmission through respiratory droplet and TB treatability by modern drugs than traditional medicine. On the other hand 85.7%, 70%, 35.5% of pharmacy, ML and other HWs working in DTH reported cigarette smoking, dust and food shortage as both risk factor and causative agent of TB respectively. Moreover there was statistically significant difference (Fisher’s exact test P = 0.004) in knowing two case finding strategies among other HWs who worked in DTH (93.6%) and HCs (60.9%). On the other hand there was no a statistical difference among ML workers working in DTH (40%) and HCs (45.5%) as well as pharmacy workers working in DTH (71.4%) and HCs (66.7%) with Chi square P = 0.768 and Fisher’s exact test P = 0.594 respectively (Table 5).

**Table 5:** Tuberculosis knowledge of health care providers working in Debre Tabor town public health facilities from January to May 2018.

No	Items used	DTH: Number responded yes (%)			HCs: Number responded yes (%)		
		Other HW	Lab	Phar	Other HW	Lab	Phar
1	**TB causative agent**						
	Caused by bacteria	31 (100)	20 (100)	21 (100)	23 (100)	10 (90.1)	6 (100)
	Caused by cold air	3 (9.7)	4 (20)	9 (42.9)	8 (34.8)	3 (27.3)	3 (50)
	Caused by Food shortage	11 (35.5)	14 (70)	16 (76.2)	20 (87)	9 (81.8)	6 (100)
	Caused by cigarette smoking	11 (35.5)	14 (70)	18 (85.7)	20 (87)	8 (72.7)	5 (83.3)
	Caused by sunlight	2 (6.5)	6 (30)	7 (33.3)	3 (13)	5 (45.5)	2 (33.3)
	Caused by dust	11 (35.5)	14 (70)	20 (95.2)	20 (87)	9 (81.8)	5 (83.3)
	Caused by different viruses	5 (16.1)	6 (30)	11 (52.4)	12 (52.2)	5 (45.5)	5 (83.3)
	Have genetic cause	2 (6.5)	1 (5)	0	2 (8.7)	0	0
3	**TB diagnosis & follow up**						
	Know PPos TB sputum follow up	15 (48.4)	8 (40)	0	7 (30.4)	8 (72.7)	0
	Role of HEW in treatment follow up	26 (83.9)	0	0	1 (4.3)	1 (9)	0
4	**TB treatment**						
	Know TB drug category	**-**	**-**	12 (57.1)	**-**	**-**	2 (33)
	Know major & minor side effects	**-**	**-**	8 (30.1)	**-**	**-**	5 (83.3)
	Treated by modern drug only	30 (96.8)	20 (100)	21 (100)	23 (100)	11 (100)	6 (100)
	Treated by traditional medicine only	1 (3.2)	0	0	0	0	0
5	**TB case finding strategy**						
	Know ≥2 case finding strategy	29 (93.5)	8 (40)	15 (71.4)	14 (60.9)	5 (45.5)	4 (66.7)

Lab→ Medical Laboratory workers, Phar→ Pharmacy workers, Other HW→ Other Health Workers, PPos TB→ Pulmonary positive TB, HCs→ Health centers (Debre Tabor, Hidar 11 and Ginbot 20 health enters), HEW→ Health Extension Worker

Generally all public HFs had poor quality of care delivered in the process aspects even though there were variations among different units of each HF (Table 6).

**Table 6:** The score of process aspect of quality of care delivered for TB patients in four public health facilities of Debre Tabor town from January to May 2018.

No	HF	Unit	Process	Status	Process	Status
			% by unit (HW No/item)		% by facility (HW No/item)	
1	DTH	Laboratory	67.4 (20/23)	Poor	68.9 (72/74)	Poor
		Pharmacy	54.5 (21/28)	VP		
		OPD	80.6 (31/23)	Good		
2	DTHC	Laboratory	63.8 (3/23)	Poor	60 (15/74)	Poor
		Pharmacy	59 (2/28)	VP		
		OPD	59.1 (10/23)	VP		
3	Ginbot 20 HC	Laboratory	60.8 (4/23)	Poor	60.5 (11/74)	Poor
		Pharmacy	64.3 (2/28)	Poor		
		OPD	58.3 (5/23)	VP		
4	Hidar 11 HC	Laboratory	71.7 (4/23)	Moderate	65 (14/74)	Poor
		Pharmacy	53.6 (2/28)	VP		
		OPD	64.7 (8/23)	Poor		

DTH→ Debre Tabor Hospital, DTHC→ Debre Tabor Health Center, HW→ Health Workers, HF→ Health Facility

### Outcome aspects of quality

Based on the two year document review done to assess outcome attributes of quality of care delivered for TB patients, a total of 354 patients had attended their treatment in four public HFs studied of which 188 (53.1%) of them were male and 166 (46.9%) females where DTH accounts the highest TB patients (39.8%) from the total. In all HFs studied, all TB patients’ unit TB registration number, sex, age, TB category, treatment initiation date and intensive phase treatment start year is properly registered. Furthermore 110 (78%) and 147 (69%) contact person address in DTH and HCs was properly registered on TB unit register book respectively with no statistical difference in hospital and HCs (P = 0.063). On the other hand 97 (27.4%) and 144 (40.7%) contact person address and TB patient address were not properly indicated on TB unit register book respectively. There were statistically significant (P = 0.000) proper registration of TB patient contact address in DTH (83%) than HCs (43.7%). The study indicated that the overall outcome aspect of quality of care delivered for TB patients in HFs assessed, except Ginbot 20 HC which scored moderate, were good.

## Discussion

This study assessed the three aspects of quality of TB care delivered for TB patients in public HFs of Debre Tabor town.

### Structure aspect of quality

The present study indicated that all HFs assessed had sufficient anti TB drug supply for 3 months. This finding is in agreements with four studies done in Uganda [12], Nigeria [11], Ethiopia (Jimma [7] and Bahir Dar [10]). On the other hand 92.6% of pharmacy workers in the present study reported that their HF make drugs available in weekend Which was higher than a study done in Jimma Zone by Taddese G et al which reported only 10% [7]. The variation may be because of study time gap and study setting difference.

Moreover all HFs had at least one functional microscope used for TB diagnostic service and separate toilet room for females and males which was slightly higher than study done in Jimma, Ethiopia which reported 70% of HFs had toilet facility [7]. All public HFs had sufficient laboratory reagent, microscopic slides for sputum microscopy and TB posters for health education which was higher than a study done in Jimma Zone, Ethiopia which reported only 60% of HFs had sufficient laboratory reagents, microscopic slides for sputum smear microscopy and 20% of HFs had TB related posters. Moreover all HFs had at least 2 HWs who had taken special training on DOTS which was higher than a study in Jimma, Ethiopia which reported only 50%. [7]. These variations might be due to study time gap where the study in Jimma zone was conducted in 2008 which is 10 years back compared to the current study since there are a lot of improvements in the health system of Ethiopia including different supplies and guidelines. On the other hand, the result of the present study on availability of reagents and microscopes agrees with a study done in Uganda by Lilian B, et al which reported that all eight HFs have reagents and microscope [12].

National TB guideline of Ethiopian recommend HF laboratories to have laboratory reagents used to diagnose TB suspects taking sputum specimen by conventional microscopic technique as a backup or complementary to fluorescent microscopy technique [4]. But the present study indicated that 1 (25%) HF hadn’t had backup or complementary staining.

The present study also indicated that all HFs have separate sputum specimen collection area which disagrees with studies done in Jimma zone, Ethiopia [7] and Kersa district, Ethiopia [9] which reported that all HFs assessed didn’t have separate sputum collection area. This can be explained by a study period gap and geographical location difference.

All HFs in the present study didn’t have a copy of the national TB guideline which agrees with a study done in Jimma zone by Taddese G et al which reported that only 10% of HFs assessed had the national guideline [7]. The low finding observed in this study might be explained that all HFs assessed had the new version of TB manual [13] developed from the national TB guideline. This result agrees with a study in Bahir Dar public HFs by Mulatu K et al in 2013 [10] where all HFs assessed had the new version of TB manual.

In the present study, all HFs had supervisory support from different stakeholders which was higher than a study in Tigray regional state of Ethiopia which reported that 34 (77.3%) HFs had supervisory support [5]. This difference could be explained that the study in Tigray included 26 clinics (which were more than half of the HFs studied) unlike present study which didn’t include clinics. On the contrary the present study finding was extremely higher compared with a study done in Bahir Dar, Ethiopia [10] where only 2 (28.6%) public HFs assessed were supervised. This variation may be due to the reason that the study done in Bahir Dar mainly assessed quality of DOTS from the perspective of patients and included only one HW from each HF. Since the supportive supervision might not be known by all HWs, it is recommended to take a representative HW number as it was being observed in the present study that 28 (33.9%) of HWs included in the study didn’t know the existence of supervision in their HFs.

Even though the present study indicated a lot of improvements (on availability of different drug supply, laboratory reagents, TB posters and DOTS training taken), the overall structural aspect of quality of care delivered was very poor which was similar with a study done in Jimma, Ethiopia [7]. These may be explained that, unlike the study in Jimma which used checklists, the present study assessed using questionnaire. Since the present study indicated that 88.4% and 54.5% of HWs interviewed were not satisfied with the current payment and management system of their HFs respectively which might trigger HWs or make them negligent to under report during interview. On the other hand, this might indicate that resource is scarce and it is still a challenge for developing countries including Ethiopia.

### Process aspect of quality

One of the best methods indicated in Ethiopian national guideline [4] for TB control program is prompt TB case finding of TB suspects. But the present study indicated different findings in knowing only 2 case finding strategies out of 8 strategies indicated in the national guideline. Twenty nine (93.6%) HWs working in DTH knew at least two case finding strategies unlike HWs working in HCs where only 23 (57.5%) know the strategy. These result indicated that most HWs didn’t know the case finding strategies stated by Ethiopian national guideline [4].

The present study also showed that 14 (51.9%) pharmacy workers know anti TB drug categories which were similar with a study done in Jimma, Ethiopia where 45% HWs knew the categories. On the other hand the present study indicated that 48.1% of pharmacy worker did know the major and minor drug side effects which were higher than a study in Jimma, Ethiopia which reported only 15% [7]. This variation might be explained by study time gap and respondent HWs difference included in percentage calculation where in the present study, only pharmacy workers were included unlike a study in Jimma which include other HWs like nurses, health assistants and others.

### Outcome aspect of quality

In the present study, all TB patients’ unit TB registration number, age and sex were properly recorded which aggress with a study done in Bahr Dar, Ethiopia [10]. On the other hand, the present study also showed that 97 (27.4%) and 144 (40.7%) contact person address and TB patient address were not properly indicated on TB unit register book respectively which was higher than a study in Jimma, Ethiopia which reported 17.5% and 23.1% of contact person address and patient address as not indicated respectively [7]. This variation might be explained that in the present study, complete patient address or contact person address was considered if patient or contact person address was written together with phone number/“kebel” address than simply patient name or contact person name presence on the TB unit register book.

## Conclusions

In the three aspects of quality of care assessed, good practices and different constraints in delivering quality care were identified. Based on the study, we concluded that all HFs have the recent TB user manual, enough microscopic slides, separate sputum collection area, sufficient drug supply, provide health education on TB, knowledge on cause of TB and better TB register book documentation which will enhance the quality of care delivered for TB patients. On the other hand, the study indicated that there were no up-to-date in-service trainings given on TB for HWs, laboratory reagent back up and participatory management and incentive system. Moreover majority of HWs have limited knowledge on TB risk factors, TB case finding strategy and TB patient follow up strategy. Over all the present study indicated that all public HFs included in the present study had very poor, poor and good (except Ginbot 20 HC) structural, process and outcome aspects of quality of care delivered for TB patients respectively.

In order to achieve different international commitments signed by Ethiopia on TB like the EndTb strategy set by WHO and sustainable development goal 2030 set by United Nations, different work shall be done on TB diagnosis, treatment and prevention. Moreover WHO EndTb strategy (up to 2035) states optimizing interventions done on TB diagnosis, treatment and prevention as one of its pillars based on different research findings [1]. Thus based on the present study findings, we recommend Woreda and Zonal health offices, regional bureau, national health ministry of Ethiopia and other stakeholders involved in the diagnosis, treatment and prevention of TB to enhance mainly on the structure and process aspects of quality of care delivered for TB patients. Moreover based on the study findings, continuous supply of drugs, laboratory equipment and reagent, availing the current guideline/s in health facilities, providing up-to-date training for HWs on TB and proper documentation are important to improve quality of care provided for TB patients.

## Abbreviations

BSc: Bachelor of Science
DOTS: Directly Observed Treatment Short course
DTH: Debre Tabor Hospital
DTHC: Debre Tabor Health Center
DTU: Debre Tabor University
HC: Health Center
HFs: Health facilities
HIV: Human Immunodeficiency Virus
HWs: Health Workers
ML: Medical Laboratory
TB: Tuberculosis
WHO: World Health Organization
